# Endocytosis of indium-tin-oxide nanoparticles by macrophages provokes pyroptosis requiring NLRP3-ASC-Caspase1 axis that can be prevented by mesenchymal stem cells

**DOI:** 10.1038/srep26162

**Published:** 2016-05-19

**Authors:** Abderrahim Naji, Basilua André Muzembo, Ken-ichi Yagyu, Nobuyasu Baba, Frédéric Deschaseaux, Luc Sensebé, Narufumi Suganuma

**Affiliations:** 1 Center for Innovative and Translational Medicine (CITM), Kochi Medical School, Kochi University, Kochi, Japan; 2 Department of Environmental Medicine, Kochi Medical School, Kochi University, Kochi, Japan; 3 Science Research Center, Division of Biological Research, Life Sciences and Functional Materials, Kochi Medical School, Kochi University, Kochi, Japan; 4 STROMALab, Université de Toulouse, UMR 5273 CNRS, INSERM U1031, EFS Pyrénées-Méditerranée, Toulouse, France; 5 Department of Epidemiology, Infectious Disease Control and Prevention, Institute of Biomedical and Health Sciences, Hiroshima University, Hiroshima, Japan

## Abstract

The biological effects of indium-tin-oxide (ITO) are of considerable importance because workers exposed to indium compounds have been diagnosed with interstitial lung disease or pulmonary alveolar proteinosis; however, the pathophysiology of these diseases is undefined. Here, mice intraperitoneally inoculated with ITO-nanoparticles (ITO-NPs) resulted in peritonitis dependent in NLRP3 inflammasome, with neutrophils recruitment and interleukin-1β (IL-1β) production. Withal peritoneal macrophages exposed *ex vivo* to ITO-NPs caused IL-1β secretion and cytolysis. Further, alveolar macrophages exposed to ITO-NPs *in vitro* showed ITO-NP endocytosis and production of tumor necrosis factor-α (TNF-α) and IL-1β, ensued cell death by cytolysis. This cell death was RIPK1-independent but caspase1-dependent, and thus identified as pyroptosis. Endocytosis of ITO-NPs by activated THP-1 cells induced pyroptosis with IL-1β/TNF-α production and cytolysis, but not in activated THP-1 cells with knockdown of NLRP3, ASC, or caspase1. However, exposing activated THP-1 cells with NLRP3 or ASC knockdown to ITO-NPs resulted in cell death but without cytolysis, with deficiency in IL-1β/TNF-α, and revealing features of apoptosis. While, mesenchymal stem cells (MSCs) co-cultured with macrophages impaired both inflammation and cell death induced by ITO-NPs. Together, our findings provide crucial insights to the pathophysiology of respiratory diseases caused by ITO particles, and identify MSCs as a potent therapeutic.

Recently, respiratory diseases were reported in workers exposed to indium compounds such as indium oxide (InO) and indium tin oxide (ITO) particles^1^. These workers showed interstitial lung diseases within 4 to 13 years after the first exposure or pulmonary alveolar proteinosis just 1 to 2 years after the first exposure. As compared with the most prevalent form of silicosis, which generally results from exposure to silica (SiO_2_) particles for 20 years or more^2^, the onset of lung diseases among workers exposed to indium compounds seems to occur promptly after exposure^1^.

Current studies have revealed that activation of NOD-like receptor (NLR), pyrin domain-containing 3 (NLRP3) inflammasome in macrophages often results in excessive inflammation responsible for various diseases^3^,^4^. As well, the development of pulmonary fibrosis after exposure to SiO_2_ particles or asbestos fibers may depend on NLRP3 inflammasome activation^5^,^6^,^7^. Sintered-ITO was suggested to cause *in vitro* adverse inflammatory responses in macrophages and epithelial cells that could involve in part inflammasome activation^8^. However, whether ITO-NPs can activate the NLRP3 inflammasome *in vivo* is unknown. Moreover, the molecular mechanisms implicated in the recognition and endocytosis of ITO-NPs by alveolar macrophages and monocyte-derived macrophages leading to caspase1-dependent inflammation and cell death are unclear.

Fundamentally, NLRP3 inflammasome is an intracellular heteromeric complex expressed in immune cells that contains the NLRP3 protein, apoptosis-associated speck-like protein containing the caspase recruitment domain (ASC) and pro-caspase1. Following NLRP3 inflammasome activation and assembly, pro-caspase1 is cleaved *via* autocatalytic processes to result in the active form of caspase1^9^. NLRP3 inflammasome is activated by diverse agents including adverse signals such as extracellular ATP *via* the purinergic P2×_7_ receptor (P2×_7_R) associated with pannexin1 (Panx1) K^+^ hemichannels^10^,^11^. In particular, NLRP3 inflammasome is activated by crystalline and particulate substances that are produced in excess by dysfunctional tissues such as monosodium urate (MSU) crystals in gout^12^, and amyloid fibrils in type-2 diabetes^13^, or inorganic xenogenous compounds such as SiO_2_ particles and asbestos fibers^6^. Activation of NLRP3 inflammasome leads to the maturation, *via* caspase1, of pro-interleukin (pro-IL-1β) and pro-IL-18 cytokines into their active and secreted forms, IL-1β and IL-18^14^. Eventually, activation of NLRP3 inflammasome results in the execution of a regulated cell death (RCD) known as pyroptosis through cleavage of Gasdermin D (GSDMD) by caspase1, which ultimately causes the loss of plasma membrane integrity^15^,^16^,^17^. In contrast to a RCD event such as apoptosis, which is non-lytic and non-immunogenic^16^, pyroptosis is completed by the rupture of the plasma membrane, with potent immunogenicity and with implications in the pathogenesis of various diseases^15^.

To initiate inflammation, particles and fibers must be detected and engulfed by macrophages and this can require surface receptors to recognize xenogenous compounds^18^. Scavenger receptors (SRs) such as SR class-A (SR-A) MARCO and CD204 or SR class-B (SR-B) CD36, have been suggested to facilitate the uptake of SiO_2_ particles and asbestos fibers by macrophages^18^,^19^,^20^,^21^,^22^. Further, CD36 directly coordinates NLRP3 inflammasome activation mediated by oxidized low-density lipoprotein (LDL), amyloid-β and amylin peptides, in sterile inflammation related-diseases such as atherosclerosis, Alzheimer’s disease and type-2 diabetes^13^. Therefore, SRs could function upstream of NLRP3 inflammasome and caspase1, which suggests significant therapeutic targets in treating respiratory diseases due to chronic inhalation of xenogenous compounds^13^,^18^,^21^,^22^.

This study is motivated to provide molecular insights to the pathophysiology of respiratory diseases caused by occupational exposure to indium compounds and to identify a therapeutic strategy for these diseases. Our hypothesis is that resident macrophages and monocyte-derived macrophages, exposed to small-sized particles of ITO induce NLRP3-ASC inflammasome activation resulting in severe inflammation and pyroptotic cell death. These conditions are particularly detrimental as they can allow a prolonged inflammation, tissue damages and fibrosis^23^,^24^. Moreover, we hypothesized that mesenchymal stem cells (MSCs), well-known for their immunosuppressive and regenerative properties^25^,^26^, could prevent both inflammation and cell death of macrophages upon exposure to ITO particles.

Therefore, this work aimed to elucidate the mechanisms of ITO-NP–mediated inflammation *in vivo* in mice and *in vitro via* extracellular recognition by SRs of ITO-NPs by alveolar macrophages to caspase1-dependent pyroptosis execution. We also aimed to examine the path of ITO-NP uptake by human monocyte-derived macrophages to intracellular NLRP3-ASC inflammasome activation and caspase1-dependent pyroptosis execution. We compared the effects of ITO-NPs in alveolar macrophages and monocyte-derived macrophages with inflammatory compounds such as silica nanoparticles (SiO_2_-NPs) and MSU crystals. Finally, we evaluated human bone marrow-derived mesenchymal stem cells (MSCs) as suppressors of pyroptosis in co-culture with alveolar macrophages and monocyte-derived macrophages exposed to ITO-NPs, SiO_2_-NPs or MSU.

Here, we report that 1) ITO-NPs induce peritonitis in mice with neutrophil recruitment and IL-1β production that depends on NLRP3 inflammasome and that, 2) endocytosis of ITO-NPs induce caspase1-dependent pyroptosis in macrophages *via* extracellular interaction with at least SR-A CD204 and caspase1 function, involving intracellular activation of NLRP3-ASC inflammasome and that, 3) MSCs act as a pyroptosis suppressor. The pyroptosis induced by ITO-NPs like SiO_2_-NPs in macrophages may be a mechanism of exacerbated inflammation in lung diseases caused by inhalation of inorganic particles such as ITO and SiO_2_, and MSCs treatment may be a potential therapeutic suppressor of the pyroptosis.

## Results

### Intraperitoneal inoculation in mice of ITO-NPs induces peritonitis dependent on NLRP3 inflammasome activation

**Figure 1:**
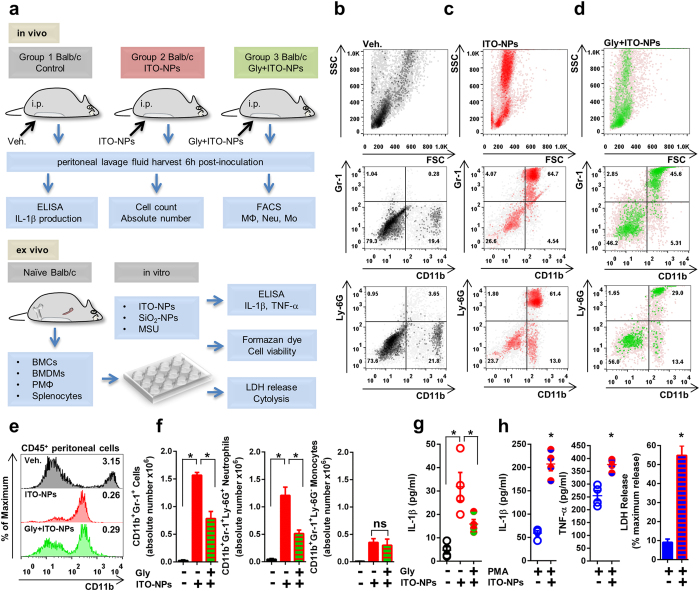
Intraperitoneal inoculation of ITO-NPs in mice induces peritonitis through NLRP3 inflammasome. (**a**) Schematic diagram of the experimental procedures used *in vivo* and *ex vivo* in Balb/c mice to examine the role of ITO-NPs in inducing NLRP3 inflammasome-dependent peritonitis. (**b**) Flow cytometer analysis of peritoneal cells of control Balb/c mice (black dots), (**c**) mice receiving ITO-NPs (red dots) and (**d**) both NLRP3 inflammasome inhibitor glybenclamide (Gly) and ITO-NPs (green dots). (**b**) Dot-plots show peritoneal cells of control mice stained for CD11b, Gr-1 and Ly-6G; about 20% of the CD45^+^ cells were peritoneal macrophages phenotypically characterized as positive for CD11b and negative for Gr-1 and Ly-6G. (**c**) 6-h post-inoculation i.p. of ITO-NPs in Balb/c mice, CD11b^+^Gr-1^+^ increases, to reach about 60% Ly-6G positive (neutrophils) and 13% Ly-6G negative (inflamed-monocytes). (**d**) Balb/c mice receiving i.p. Gly and ITO-NPs show reduced number of CD11b^+^Ly-6G^+^ neutrophils. (**b**–**d**) One representative experiment out of 4 is shown. (**e**) Offset histogram overlays representing cell surface density of CD11b antigens with mean fluorescence intensity (MFI x10^3^) on CD45^+^ peritoneal cells from control mice (black), ITO-NP–inoculated mice (red), Gly– and ITO-NP–receiving mice (green). (**f**) Absolute number of cells phenotypically characterized as CD11b^+^Gr-1^+^, CD11b^+^Gr-1^+^Ly-6G^+^ (neutrophils) and CD11b^+^Gr-1^+^Ly-6G^−^ (inflamed-monocytes) among CD45^+^ peritoneal cells from control mice and mice receiving ITO-NPs or both Gly and ITO-NPs. (**g**) IL-1β detected in peritoneal fluid from control mice, mice receiving ITO-NPs and both Gly and ITO-NPs. (**h**) Release of IL-1β, TNF-α and of LDH in mice peritoneal macrophages stimulated *ex vivo* with phorbol 12-myristate 13-acetate (PMA) with or without 500 μg/mL of ITO-NP exposure for 16 h. (**b–h**), n = 4 ± SEM, *p < 0.05. Schematic diagram abbreviations, MΦ = macrophages, Neu = neutrophils, Mo = monocytes, BMCs = bone marrow cells, BMDMs = bone marrow-derived macrophages, PMΦ = peritoneal macrophages.

MSU crystals cause an innate immune response *via* inflammasome, because mice deficient in ASC (a key component of inflammasome) show impaired neutrophil recruitment after intraperitoneal (i.p.) inoculation of MSU crystals as compared with WT mice^12^. Using a comparable approach, we found that mice with i.p. ITO-NP inoculation showed induced peritonitis with inflammatory cell recruitment (Fig. 1a–g and Supplemental Fig. S1a,b). Indeed, among CD45^+^CD11b^+^ peritoneal cells of mice inoculated i.p. with ITO-NPs, we found a marked increase of neutrophils phenotypically characterized as CD11b^+^Gr-1^+^Ly-6G^+^ and inflamed monocytes characterized as CD11b^+^Gr-1^+^ Ly-6G^−^ (Fig. 1b,c,f). Along with the efflux of neutrophils and inflamed monocytes, the level of IL-1β was significantly increased within peritoneal fluid of mice receiving ITO-NPs (Fig. 1g). By contrast, in control mice, CD45^+^CD11b^+^ peritoneal cells were negative for the Gr-1 marker, indicating an absence of neutrophils or inflamed monocytes (Fig. 1b,f). Among these peritoneal cells, a subset expressed a high density of CD11b antigens, and were further phenotypically characterized as CD11b^+^F4/80^+^ (Fig. 1e and Supplemental Fig. S1a,b), representing resting resident peritoneal macrophages^27^.

Peritonitis induced by ITO-NPs was diminished by impeding NLRP3 inflammasome activation by a sulfonylurea pharmacological inhibitor, glybenclamide (Gly) (Fig. 1d–g). Gly acts upstream of NLRP3 inflammasome by inhibiting K^+^ efflux mediated *via* the Panx1 ion channel^28^,^29^. Here, Gly greatly reduced the number of neutrophils without affecting that of inflamed monocytes in mice receiving ITO-NPs (Fig. 1c–f). Thus, the peritoneal neutrophil/monocyte cell ratio was about 4:1 in mice receiving ITO-NPs but <2:1 in mice receiving both Gly and ITO-NPs (Supplemental Fig. S1c). Furthermore, Gly restrained IL-1β production in ITO-NPs receiving mice (Fig. 1g). Here, our data indicate that ITO-NPs activate NLRP3 inflammasome *in vivo* to exacerbate inflammation.

To verify whether resident peritoneal macrophages could secrete pro-inflammatory cytokines and undergo cytolysis in response to ITO-NPs, we examined their ability *ex vivo* to secrete pro-inflammatory cytokines IL-1β and TNF-α and to release lactate dehydrogenase (LDH) after exposure to ITO-NPs. We found that peritoneal macrophages exposed to ITO-NPs resulted in IL-1β and TNF-α production and release of LDH (Fig. 1h), similar results were observed when peritoneal macrophages were exposed to SiO_2_-NPs and MSU (Supplemental Fig. S1d). As well, to exclude any endotoxin-mediated effect resulting from contamination of ITO-NPs, splenocytes and bone marrow cells (BMCs) isolated from naïve mice were exposed to ITO-NPs or lipopolysaccharide (LPS) and examined for their viability and ability to secrete TNF-α. Splenocytes and BMCs responded vigorously to LPS in terms of TNF-α production but were unable to secrete TNF-α on exposure to ITO-NPs at 50, 100 and 500 μg/mL, whilst cell viability was not disturbed for any treatments tested (Supplemental Fig. S2a,b). Thus, ITO-NPs were excluded from contamination with endotoxins and ITO-NPs could not affect splenocytes, or immature BMCs. Yet, in agreement with the findings obtained with resident peritoneal macrophages, ITO-NPs affected bone marrow-derived macrophages (BMDMs) by stimulating the release of IL-1β and TNF-α, and by decreasing cell viability through cytolysis (Supplemental Fig. S2c,d). Here, our findings indicate that ITO-NPs affect specifically macrophages. These unfavorable effects of ITO-NPs on macrophages might be related to their capability for particles uptake.

### Endocytosis of ITO-NPs by alveolar macrophages provokes TNF-α and IL-1β production and caspase1-dependent cell death

**Figure 2:**
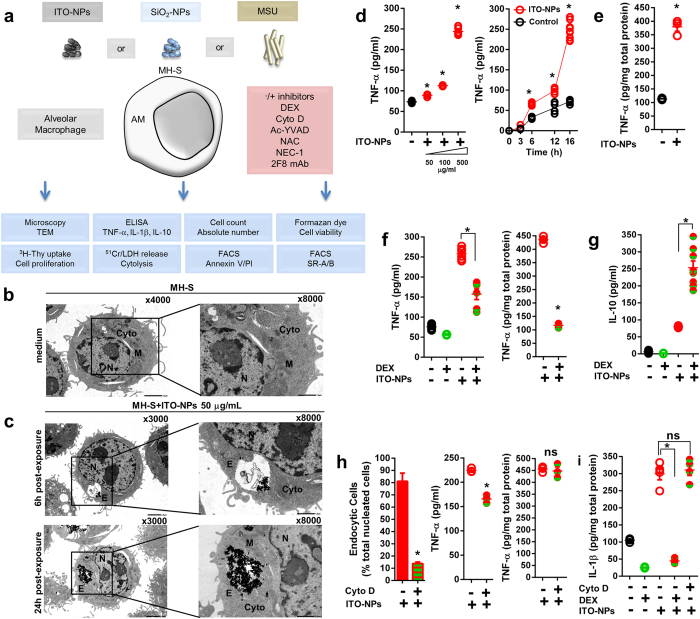
Alveolar macrophage endocytosis of ITO-NPs enhances TNF-α and IL-1β pro-inflammatory cytokine production. (**a**) Schematic diagram of the experimental procedures to consider alveolar macrophage response upon exposure to ITO-NPs, SiO_2_-NPs or MSU. (**b**) TEM of non-exposed MH-S cells shows preserved cytoplasmic organelles, regular microvilli protruding, and well-delineated nucleus with heterochromatin. (**c**) MH-S cells exposed for 6 h to 50 μg/mL ITO-NPs exhibited early endosomes containing ITO-NPs appearing as dark spots. At 24 h post-exposure to ITO-NPs at 50 μg/mL, endosomes in MH-S contained an increased amount of ITO-NPs. **(b,c)** Original magnification x3000, x4000 and x8000, 1 representative TEM image is shown from at least 3. (**d**) MH-S cell production of TNF-α upon exposure to increasing doses, i.e. 50, 100 and 500 μg/mL, of ITO-NPs. MH-S cells exposed to ITO-NPs at 500 μg/mL and examined at 3, 6, 12 and 16 h for secretion of TNF-α. (**e**) Intracellular TNF-α level found in MH-S cells exposed ITO-NPs compared to unstimulated MH-S. (**f**) Secreted and intracellular TNF-α levels in MH-S cells treated with dexamethasone (DEX) and exposed to ITO-NPs. (**g**) IL-10 production in MH-S cells exposed to ITO-NPs with DEX treatment and compared to controls. (**h**) MH-S cells treated with or without Cyto D and exposed to ITO-NPs for 12 h were analyzed in 3 representative areas (RA) on x500 TEM images. The number of endocytic cells containing ITO-NPs was compared to the total number of nucleated cells in RA; data are given as percentage of endocytic cells. Cyto D effect was evaluated for TNF-α secretion and intracellular TNF-α expression in MH-S upon exposure to ITO-NPs. (**i**) Intracellular IL-1β level was evaluated in MH-S cells with ITO-NP exposure; with or without DEX and Cyto D treatment compared to controls. The concentration of ITO-NPs was 500 μg/mL and time of exposure 16 h, unless stated otherwise. (**b–i**) n = 3–8 ± SEM, *p < 0.05. TEM abbreviations, Cyto = cytoplasm, N = nucleus, M = mitochondria and E = endosome.

Next, we compared the molecular mechanisms leading to inflammation and cell death in alveolar macrophages (AMs) exposed to ITO-NPs, SiO_2_-NPs and MSU crystals (Fig. 2a). Dysfunction of AMs is often associated with the pathogenesis of respiratory diseases^30^. Particularly, AMs prevent xenogenous compounds from accumulating into alveolar sacs, and AM endocytic potentials provide a vital defense^30^. We used transmission electronic microscopy (TEM) to investigate the uptake of ITO-NPs by MH-S cells, an established murine cell line of AMs. At 6-h post-exposure to 50 μg/mL ITO-NPs, MH-S cells exhibited endosomes containing ITO-NPs appearing as aggregated electronic dense spots (Fig. 2b,c). At 24-h post-exposure, endosomes were enlarged and contained an increased amount of ITO-NPs (Fig. 2c). With higher concentrations of ITO-NPs, MH-S cells showed endosomes comprising an augmented amount of ITO-NPs (Supplemental Fig. S3). Thus, the size of endosomes containing ITO-NPs was dose and time-dependent. As well, MH-S cells stimulated with LPS and exposed to ITO-NPs exhibited endosomes containing ITO-NPs (Supplemental Fig. S4a). Additionally, MH-S cells were capable of uptake of SiO_2_-NPs and MSU crystals (Supplemental Fig. S4b), compounds with a strong ability to trigger activation of NLRP3 inflammasome^14^. MH-S cells exposed to ITO-NPs dose- and time-dependently secreted TNF-α (Fig. 2d). In parallel, intracellular TNF-α level was increased (Fig. 2e), which demonstrates *de novo* protein production by macrophages in response to ITO-NPs. As well, intracellular and secreted TNF-α level was increased in MH-S cells exposed to SiO_2_-NPs or MSU crystals (Supplemental Fig. S4d–f).

Further, we examined dexamethasone (DEX) as a potent immunosuppressive molecule that could prevent ITO-NPs–induced inflammatory signaling in macrophages. DEX diminished the intracellular and secreted TNF-α level in MH-S cells exposed to ITO-NPs (Fig. 2f), but secretion of the immunosuppressive cytokine IL-10 was increased (Fig. 2g). As well, DEX lessened TNF-α secretion in MH-S cells exposed to SiO_2_-NPs (Supplemental Fig. S4e). Next, we examined ITO-NP uptake blockade by using an inhibitor of actin polymerization, cytochalasin D (Cyto D). Cyto D prevented endocytosis of ITO-NPs in MH-S cells (Fig. 2h and Supplemental Fig. S4c), and significantly decreased TNF-α secretion (Fig. 2h). However, and by contrast to DEX, Cyto D did not counter the intracellular overexpression of TNF-α in MH-S cells exposed to ITO-NPs (Fig. 2h). As compared with Cyto D, DEX may regulate inflammatory proteins at the transcriptional level in preventing inflammatory signaling induced by ITO-NPs. In accordance with this data, MH-S cells exposed to ITO-NPs or SiO_2_-NPs showed upregulated IL-1β expression (Fig. 2i and Supplemental Fig. S4g), and DEX but not Cyto D markedly inhibited IL-1β overexpression (Fig. 2i). Here, blockade of endocytosis indicated that ITO-NPs could interact with surface receptors expressed on AMs, which was sufficient to transduce intracellular pro-inflammatory signaling. Moreover, DEX might counteract these inflammatory signaling at the transcriptional level.

### ITO-NPs induce caspase1-dependent pyroptosis in alveolar macrophages involving scavenger receptor class-A (SR-A) CD204

**Figure 3:**
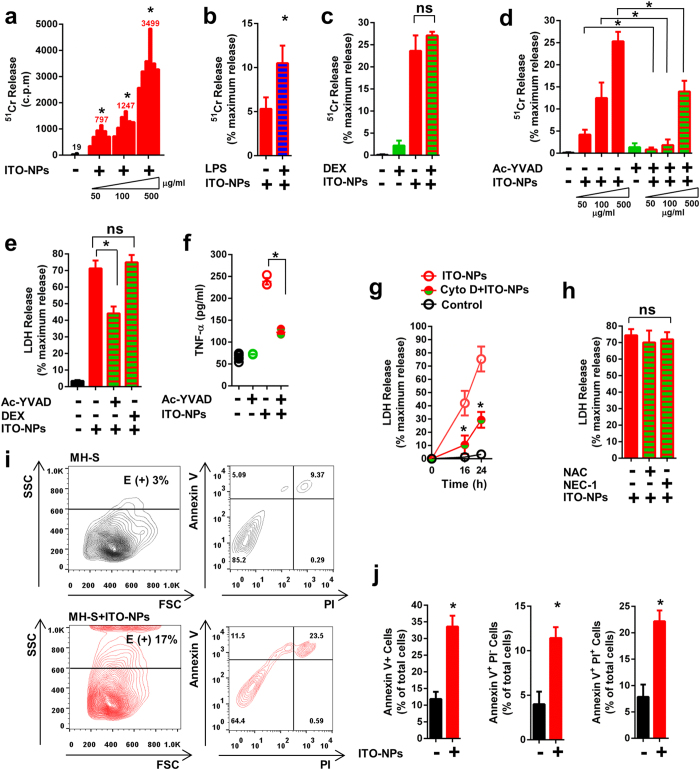
ITO-NPs endocytosis by alveolar macrophages elicits caspase1-dependent pyroptosis. (**a**) MH-S cell release of ^51^Cr increased in dose-dependently on exposure to 50, 100, and 500 μg/mL of ITO-NPs. (**b**) MH-S cells treated with LPS and exposed to 50 μg/mL ITO-NPs were compared to control for release of ^51^Cr. (**c**) DEX treated MH-S were evaluated for ^51^Cr release on exposure to ITO-NPs. (**d**) Blockade of caspase1 with Ac-YVAD in MH-S cells exposed to increasing doses of ITO-NPs were evaluated for preventing ^51^Cr release. (**e**) LDH release was examined in MH-S cells exposed to 500 μg/mL ITO-NPs for 24 h with Ac-YVAD or DEX treatment. (**f**) TNF-α secretion from MH-S cells exposed to 500 μg/mL ITO-NPs with or without Ac-YVAD, 16 h post-exposure, were measured and compared to controls. (**g**) The effect of ITO-NPs endocytosis blockade with Cyto D were evaluated in MH-S cells for LDH release at 16 and 24 h post-exposure to 500 μg/mL ITO-NPs. (**h**) ROS scavenger N-acetyl-(L)-cysteine (NAC) and blockade of RIPK1 with necrostatin-1 (NEC-1) were tested in MH-S cells at 24 h post-exposure to 500 μg/mL ITO-NPs for LDH release. (**i**) MH-S cells exposed to ITO-NPs for 8 h compared to unstimulated MH-S were analyzed after Annexin V and propidium iodide (PI) staining on flow cytometer. Dot plot of Annexin V and PI staining of MH-S cells exposed to ITO-NPs (red) compared to control (black). FSC/SSC shows MH-S with ITO-NPs uptake with increasing granularity compared to control and cells noted E(+) are for cells containing endosome. Percentage of E(+) cells are given. (**j**) Percentage of total cells being stained with Annexin V and PI are given. An increase in the percentage of cells double positive Annexin V^+^PI^+^ among MH-S exposed to ITO-NPs, suggest a cell death with plasma membrane rupture. (**a–j**) n = 3–6 ± SEM, *p < 0.05.

We next investigated ITO-NP–mediated cell death in AMs and whether caspase1, endocytosis and SRs are involved in this process. In addition to increasing pro-inflammatory cytokine production, ITO-NPs inhibited cell proliferation of MH-S cells (Supplemental Fig. S5a) and, LPS exacerbated this inhibition (Supplemental Fig. S5b). DEX did not prevent cell proliferation inhibition in MH-S exposed to ITO-NPs (Supplemental Fig. S5c). This cell proliferation inhibition was accompanied by a decrease in cell viability (Supplemental Fig. S5d,g), and consistently, LPS intensified the decreased cell viability (Supplemental Fig. S5e,h). Of note, DEX could not reverse the loss of cell viability induced by ITO-NPs in MH-S cells (Supplemental Fig. S5f,i). Furthermore, we examined the ability of ^51^Cr-radiolabeled MH-S cells exposed to ITO-NPs to release ^51^Cr into the extracellular milieu, which represents a direct measurement of AM plasma membrane rupture. ^51^Cr-radiolabeled MH-S exposed to ITO-NPs dose-dependently released ^51^Cr into the extracellular milieu (Fig. 3a), and LPS augmented the ^51^Cr release (Fig. 3b). Of note, DEX did not prevent MH-S cells from releasing ^51^Cr into the extracellular milieu with ITO-NP exposure (Fig. 3c). On the other hand, pharmacological blockade of caspase1 activity by Ac-YVAD significantly reduced ^51^Cr release resulting from exposure of ^51^Cr-radiolabeled MH-S cells to ITO-NPs (Fig. 3d). Consistently, exposed MH-S cells released LDH; Ac-YVAD but not DEX prevented the LDH release (Fig. 3e). Ac-YVAD diminished TNF-α secretion of exposed MH-S cells (Fig. 3f). Together, these data reveal that AMs exposed to ITO-NPs undergo a regulated cell death (RCD), with plasma membrane rupture, dependent on caspase1 that is evocative of pyroptosis^31^.

Next, we sought to precise the molecular pathway involved in AM cell death induced by ITO-NPs. Cyto D prevented the LDH release in MH-S exposed to ITO-NPs (Fig. 3g), which demonstrates a critical role for ITO-NP endocytosis in triggering pyroptosis. Comparable protective effects of Cyto D were observed in MH-S cells exposed to SiO_2_-NPs (Supplemental Fig. S6a,b) or MSU crystals (Supplemental Fig. S6c,d). Furthermore, we examined whether necroptosis, a RCD that depends on receptor-interacting Ser/Thr-protein kinase 1 (RIPK1)^32^, or accidental cell membranes rupture due to oxygen species overproduction^33^, are involved in ITO-NPs–induced cell death. Necrostatin-1 (NEC-1), a potent RIPK1 inhibitor, did not neutralize LDH release in MH-S exposed to ITO-NPs (Fig. 3h), nor did neutralizing reactive oxygen species (ROS) with N-acetyl-(L)-cysteine (NAC), a ROS scavenger, diminish cell death by plasma membrane rupture in exposed MH-S cells (Fig. 3h). These data highlight a central role for caspase1 in inducing a RCD in AMs exposed to ITO-NPs, and excludes a cell death *via* RIPK1-mediated necroptosis or a cell death resulting from cell membranes damage due to ROS overproduction. Further, MH-S cells exposed to ITO-NPs weakly exhibited phosphatidylserine (PS) phospholipids on their outer leaflet membrane as revealed by a modest increase in the number of AnnexinV^+^PI^−^ cells (Fig. 3i,j). Whereas, AnnexinV^+^PI^+^ cells were further represented in MH-S exposed to ITO-NPs (Fig. 3i,j), indicating breakage in plasma membrane integrity and featuring pyroptotic cells. These data suggest a RCD occurring in MH-S cells on ITO-NP exposure that is not necroptosis nor apoptosis, but a RCD caspase1-dependent, with plasma membrane disruption, typical of pyroptosis^16^.

**Figure 4:**
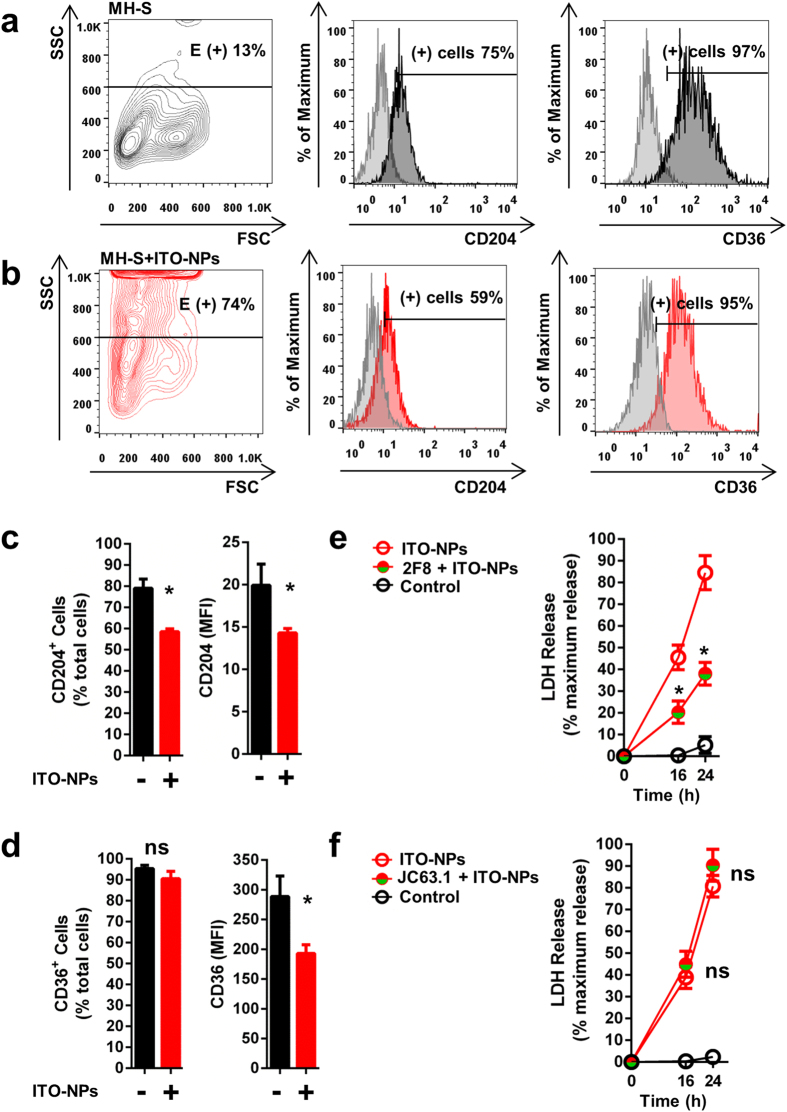
Blockade of CD204 but not CD36 counteracts pyroptosis in alveolar macrophages exposed to ITO-NPs. (**a**) Cell surface expression in resting MH-S of SR-A (collagenous) CD204 and SR-B (non-collagenous) CD36, was assessed by flow cytometer analysis (dark grey). Histograms in light grey represent isotype control. (**b**) Flow cytometer analysis (red) of CD204 and CD36 surface expression in MH-S cells exposed to ITO-NPs. (**a,b**) FSC/SSC dot-plots show MH-S with ITO-NPs uptake with increasing granularity compared to control and cells noted E(+) represented cells with endosomes. Percentage of E(+) cells are given. (**c**) Histograms showing the percentage of CD204^+^ cells and their respective MFI in unstimulated MH-S cells and MH-S cells exposed to ITO-NPs. (**d**) Percentage of CD36^+^ and their respective MFI in unstimulated MH-S cells compared to MH-S cells exposed to ITO-NPs. (**e**) Blockade of CD204 using purified monoclonal antibodies (mAbs) clone 2F8 (anti-CD204) or (**f**) blockade of SR-B CD36 with mAbs clone JC63.1 (anti-CD36) in MH-S cells exposed to ITO-NPs. (**e,f**) MH-S cells were examined for LDH release at 16 and 24-h post-exposure to ITO-NPs compared to controls. Concentration of ITO-NPs was 500 μg/mL, time of exposure was 16 h unless stated otherwise. n = 4–6 ± SEM, *p < 0.05.

To identify the receptors expressed by AMs that could enable ITO-NPs to induce pyroptosis, we first examined whether MH-S cells could express the collagenous scavenger receptor class-A (SR-A) MARCO, CD204 and the non-collagenous SR-B CD36 and whether ITO-NPs could interact with SRs. MH-S cells expressed SR-A CD204 and SR-B CD36, but SR-A MARCO were almost not detectable on cell surface (Fig. 4a and Supplemental Fig. S7a). Furthermore, MH-S cells exposed to ITO-NPs and SiO_2_-NPs showed a marked decrease in SR-A CD204 surface expression and to a lesser extent SR-B CD36 expression (Fig. 4a–d and Supplemental Fig. S7b–e). Hence, we examined whether neutralizing SR-A MARCO, CD204 or SR-B CD36 with specific monoclonal antibodies (mAbs) could reverse ITO-NP– and SiO_2_-NP–induced pyroptosis. Remarkably, blockade of SR-A CD204 with 2F8 mAb significantly prevented the death of MH-S cells exposed to ITO-NPs or SiO_2_-NPs as measured by LDH release (Fig. 4e and Supplemental Fig. S7f). However, blockade of SR-B CD36 or SR-A MARCO with JC63.1 or ED31 mAbs, respectively, did not prevent plasma membrane disruption of MH-S cells exposed to ITO-NPs and SiO_2_-NPs (Fig. 4f and Supplemental Fig. S7g–i). Thus, our data indicate that ITO-NPs may interact with AMs through SR-A CD204 leading ultimately to caspase1-dependent pyroptosis.

### Endocytosis of ITO-NPs by phorbol 12-myristate 13-acetate (PMA)-activated monocytes is required for caspase1-dependent pyroptosis execution

**Figure 5:**
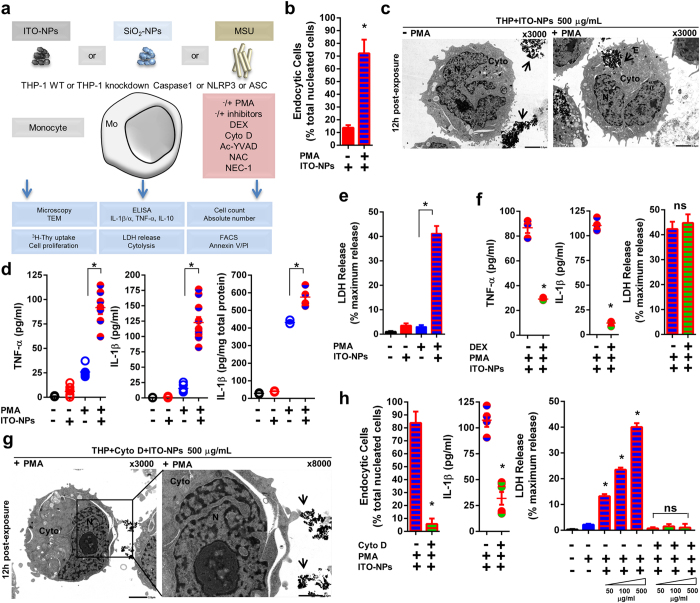
PMA-activated monocyte endocytosis of ITO-NPs results in TNF-α and IL-1β production and cytolysis. (**a**) Schematic diagram indicating the experimental procedures used to examine monocyte (THP-1 WT) and knockdown monocyte (THP-1 genetically deficient) for Caspase1 or NLRP3 or ASC response upon exposure to ITO-NPs, SiO_2_-NPs or MSU. (**b**) THP-1 cells (−) or (+) PMA uptake capability of ITO-NPs was evaluated by TEM. TEM analysis shows that activation of THP-1 cells with PMA potentiates endocytosis of ITO-NPs. Percentages of phagocytic cells are given for (−) or (+) PMA conditions in THP-1 cells exposed to ITO-NPs at 12 h post-exposure. **c,** TEM of ITO-NPs present outside (black arrow) THP-1 cells with non-activated (−) PMA compared to (+) PMA showing an endosome containing ITO-NPs; 1 representative TEM image in at least 3 is shown, original magnification ×3000. (**d**) Production of TNF-α and IL-1β and (**e**) release of LDH by PMA-activated THP-1 cells exposed to ITO-NPs compared to controls. (**d,e**) PMA potentiates THP-1 intracellular expression of IL-1β; further exposure of PMA-activated THP-1 cells with ITO-NPs augments IL-1β production and release of LDH. (**f**) DEX effects in PMA-activated THP-1 exposed to ITO-NPs in terms of TNF-α, IL-1β and LDH release. While DEX impairs TNF-α and IL-1β production, DEX does not prevent cytolysis. (**g**) Cyto D blockade of ITO-NP uptake (black arrow) by PMA-activated THP-1 cells at 12 h post-exposure; 1 representative TEM image in at least 3 is shown. (**h**) Mean percentages of endocytic cells at 12 h post-exposure to ITO-NPs are given for PMA-activated THP-1 with or without cyto D; and effects of ITO-NPs endocytosis blockade in PMA-activated THP-1 in production of IL-1β and LDH release. Overall, concentration of ITO-NPs was 500 μg/mL and time of exposure was 16 h, except if stated otherwise. (**b–h**) n = 3–9 ± SEM, *p < 0.05. TEM abbreviations, Cyto = cytoplasm, N = nucleus and E = endosome.

We next investigated the ITO-NP effects in human monocyte-derived macrophages (Fig. 5a). Cell proliferation of non-activated THP-1 cells was poorly affected by ITO-NPs (Supplemental Fig. S8a). However, when THP-1 cells were activated with PMA, cell proliferation was significantly inhibited (Supplemental Fig. S8b). THP-1 cells lacked ITO-NP uptake capability as assessed by TEM (Fig. 5b,c). However, when activated with PMA, THP-1 cell ability for endocytosis of ITO-NPs was greatly increased (Fig. 5b,c), which resulted in increased production of TNF-α and IL-1β (Fig. 5d), and ultimately cell death *via* cytolysis as assessed by LDH release (Fig. 5e). Similar results were found with PMA-activated THP-1 cells exposed to SiO_2_-NPs or MSU crystals (Supplemental Fig. S8c–e).

**Figure 6:**
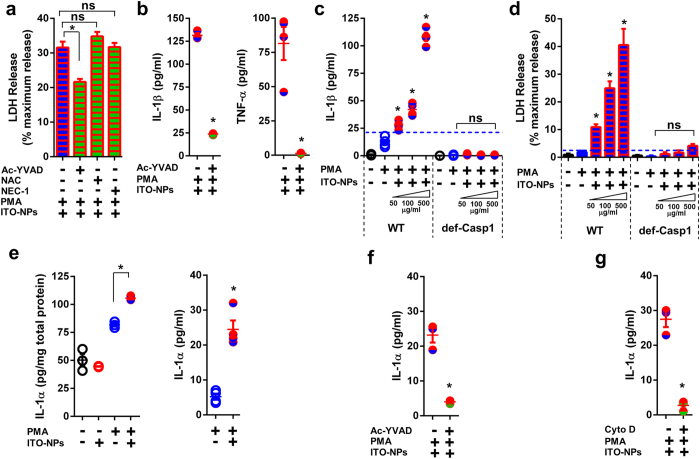
PMA-activated monocyte endocytosis of ITO-NPs induces caspase1-dependent pyroptosis. (**a**) LDH release as measured in PMA-activated THP-1 cells exposed to ITO-NPs with caspase1 activity blockade using Ac-YVAD, compared with neutralizing ROS with NAC or RIPK1 with NEC-1 (**b**) IL-1β and TNF-α secretion in PMA-activated THP-1 cells exposed to ITO-NPs with caspase1 activity blockade using Ac-YVAD. (**c**) Effect of increasing doses of ITO-NPs in PMA-activated THP-1 with a stable knockdown in caspase1 (def-Casp1) compared to wild-type (WT) PMA-activated THP-1 in terms of IL-1β secretion and (**d**) LDH release. (**e**) Intracellular expression and release of IL-1α alarmin in PMA-activated THP-1 exposed to ITO-NPs compared to controls. (**f**) Caspase1 activity blockade with Ac-YVAD or (**g**) endocytosis of ITO-NPs blockade with Cyto D, reduced IL-1α release by PMA-activated THP-1 cells exposed to ITO-NPs. Concentration of ITO-NPs was 500 μg/mL, and time of exposure was 16 h, unless stated otherwise. (**a–g**), n = 3–4 ± SEM, *p < 0.05.

Then, we considered caspase1-dependent pyroptosis in activated monocytes, to elucidate the molecular pathway of pro-inflammatory cytokines production and LDH release following ITO-NPs endocytosis. As described for AMs, DEX inhibited production of both TNF-α and IL-1β in PMA-activated THP-1 cells exposed to ITO-NPs but did not prevent cell death (Fig. 5f). Importantly, Cyto D obstructed endocytosis of ITO-NPs in PMA-activated THP-1 cells, restrained IL-1β secretion and prevented LDH release (Fig. 5g,h). By contrast, NAC and NEC-1 did not impede cell death in PMA-activated THP-1 cells exposed to ITO-NPs (Fig. 6a). Consistent with our findings regarding AMs, pharmacological blockade of caspase1 activity using Ac-YVAD, significantly restricted cell death and diminished IL-1β and TNF-α secretion in PMA-activated THP-1 cells exposed to ITO-NPs (Fig. 6a,b). Similar beneficial effect of Ac-YVAD was found in PMA-activated THP-1 cells with SiO_2_-NP or MSU crystal exposure (Supplemental Fig. S9a–b). To reveal whether caspase1 is specifically indispensable to pyroptosis in activated-monocytes ensuing ITO-NPs endocytosis, and to exclude a role of caspase4 and caspase5 in participating in this event^17^, we examined the response of THP-1 genetically knockdown for caspase1 (i.e. deficient in caspase1, abridged def-Casp1) exposed to ITO-NPs. We found that while PMA-activated wild-type (WT) THP-1 exposed to increasing doses of ITO-NPs produced IL-1β in a dose-dependent manner, PMA-activated THP-1 knockdown for caspase1 were unable to secrete IL-1β (Fig. 6c). As well, PMA-activated THP-1 caspase1 knockdown were prevented from plasma membrane rupture resulting of ITO-NPs exposure, as assessed by an absence of LDH release compared to WT control (Fig. 6d).

Thereby the pyroptosis is achieved through plasma-membrane rupture, and thus of the release of intracellular contents that can include molecules such as alarmin IL-1α^34^,^35^. IL-1α is considered to have a potent role in inducing tissue damage and fibrosis^34^,^36^. Therefore, we examined the ability of activated monocytes exposed to ITO-NPs to produce and release IL-1α. IL-1α production and release was significantly upregulated in PMA-activated THP-1 cells exposed to ITO-NPs, and similar results were found with SiO_2_-NP or MSU crystal exposure (Fig. 6E and Supplemental Fig. S9c,d). Impeding caspase1 activity or endocytosis with Ac-YVAD or Cyto D prevented IL-1α release of PMA-activated THP-1 cells exposed to ITO-NPs (Fig. 6f,g). These findings indicate that both the release of IL-1α and the cell death through cytolysis, induced by ITO-NPs in macrophages, is dependent on activity of caspase1. As well, endocytosis of ITO-NPs by PMA-activated THP-1 cells was required for IL-1α release, which is consistent with our data showing that blockade of endocytosis, prevented the rupture of plasma membrane in macrophages exposed to ITO-NPs, thus impeding intracellular content release of IL-1α.

### PMA-activated monocytes with NLRP3 or ASC knockdown show ITO-NP uptake with apoptotic cell death rather than pyroptosis

**Figure 7:**
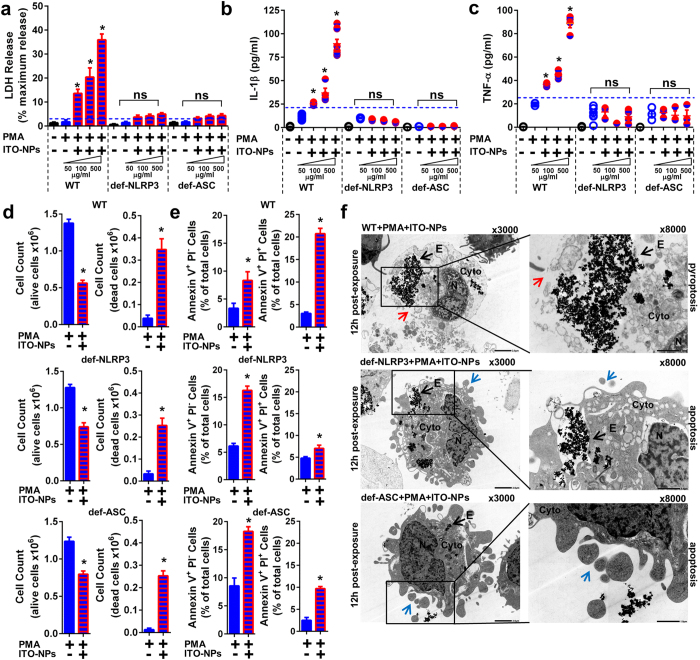
Endocytosis of ITO-NPs by PMA-activated THP-1 cells with NLRP3 or ASC knockdown does not elicit pyroptosis but rather apoptosis. (**a**) LDH release capabilities as measured in PMA-activated THP-1 either wild-type (WT) or NLRP3 knockdown (def-NLRP3), or ASC knockdown (def-ASC) on exposure to increasing doses of ITO-NPs. (**b**) PMA-activated THP-1 WT cells exposed to increasing doses of ITO-NPs but not cells with NLRP3 knockdown or ASC knockdown released IL-1β dose-dependently. (**c**) Similarly, PMA-activated THP-1 WT cells exposed to increasing doses of ITO-NPs but not cells with NLRP3 knockdown or ASC knockdown released TNF-α dose-dependently. (**d**) PMA-activated THP-1 WT cells or with NLRP3 knockdown or ASC knockdown exposed to ITO-NPs were assessed for cell viability by trypan blue dye exclusion cell count on light microscope. Results are given as number of cells alive and dead cells (×10^6^) with or without exposure to ITO-NPs. (**e**) PMA-activated THP-1 WT cells or cells with NLRP3 knockdown or ASC knockdown exposed to ITO-NPs were stained with Annexin V and PI to determine the relative number of single positive cells Annexin V^+^PI^−^ (apoptotic cells) compared to the number of cells double positive Annexin V^+^PI^+^ (pyroptotic cells). Results are given as a percentage of total cells with or without exposure to ITO-NPs. (**f**) TEM analysis of PMA-activated THP-1 WT cells exposed to ITO-NPs exhibited almost exclusively endocytic cells containing ITO-NPs with plasma membrane being ruptured; black arrow shows ITO-NPs being released after plasma membrane has dislocated (red arrow). By contrast, PMA-activated THP-1 cells with NLRP3 knockdown or ASC knockdown exhibited cells with capabilities of ITO-NPs uptake (black arrow), showing morphological features of apoptosis, such as membrane blebs and apoptotic bodies (blue arrow). TEM original magnification x3000 and x8000; 1 representative image is shown in at least 3. Concentration of ITO-NPs was 500 μg/mL, and time of exposure was 16 h, unless stated otherwise. (**a**–**f**) n = 3–8 ± SEM, *p < 0.05. TEM abbreviations, Cyto = cytoplasm, N = nucleus and E = endosome.

Because NOD-like receptor NLRP3 and its adaptor ASC are critical components of the NLRP3 inflammasome permitting caspase1 activation^4^,^14^,^37^, we next investigated whether ITO-NPs could specifically require NLRP3-ASC-Caspase1 axis for inducing pyroptosis in macrophages. We compared the effects of ITO-NPs in THP-1 cells with stable knockdown of NLRP3 (def-NLRP3) or ASC (def-ASC) and WT THP-1 cells. On exposure to ITO-NPs, PMA-activated THP-1 cells with NLRP3 or ASC deficiency were unable to release LDH, thus showing for unaltered plasma membranes (Fig. 7a). Furthermore, PMA-activated THP-1 WT cells secreted IL-1β and TNF-α with increasing doses of ITO-NPs (Fig. 7b,c); whereas PMA-activated THP-1 cells with NLRP3 or ASC deficiency were unable to secrete IL-1β or TNF-α (Fig. 7b,c).

Nonetheless, ITO-NPs increased cell death among both THP-1 WT and NLRP3 or ASC knockdown THP-1 cells with PMA activation, as assessed by optical microscope cell count on trypan blue dye exclusion (Fig. 7d). As cell viability decline was not associated with LDH release in NLRP3 or ASC knockdown THP-1 cells with PMA activation and exposed to ITO-NPs (Fig. 7a), we examined whether these cells were subjected to apoptosis. We found that WT PMA-activated THP-1 cells, upon exposure to ITO-NPs, exhibited a modest increase in AnnexinV^+^PI^−^ apoptotic cells as compared to Annexin V^+^PI^+^ pyroptotic cells (Fig. 7e). On the contrary, NLRP3 or ASC knockdown THP-1 cells with PMA activation, ITO-NP exposure resulted in a marked increase of Annexin V^+^PI^−^ apoptotic cells, while Annexin V^+^PI^+^ pyroptotic cells were not augmented compared to WT control (Fig. 7e). Consistently, TEM of PMA-activated THP-1 cells with NLRP3 or ASC deficiency exposed to ITO-NPs were capable of ITO-NPs uptake, and often exhibited apoptotic morphological features among macrophages with endosomes containing ITO-NPs, such as membrane blebs and apoptotic bodies (Fig. 7f). Conversely, among PMA-activated THP-1 WT cells exposed to ITO-NPs, unfit endocytic cells often appeared with disrupted plasma membranes (Fig. 7f). Our data suggest that circumvention of NLRP3 or ASC inflammasome components in macrophages exposed to ITO-NPs could prevent pyroptosis but facilitate alternatively apoptosis. Moreover, ITO-NP endocytosis by macrophages did not directly or, indirectly through ROS overproduction, causes damage to cell membranes, but rather demonstrated that NLRP3 inflammasome activation and caspase1 function are specifically required for pyroptosis. Blockade of inflammation using DEX or even specific blockade of NLRP3 inflammasome components might not be optimal in preventing cell death in macrophages exposed to ITO-NPs.

### Mesenchymal stem cells prevent pyroptosis in alveolar macrophages and activated monocytes with ITO-NP, SiO_2_-NP or MSU crystal exposure

**Figure 8:**
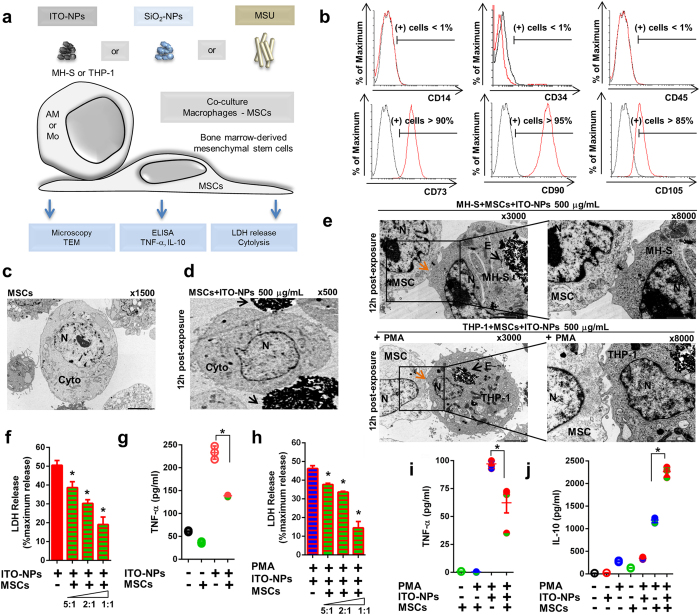
Mesenchymal stem cells (MSCs) prevent pyroptosis in macrophages with ITO-NP, SiO_2_-NP and MSU exposure. (**a**) Schematic diagram indicating the experimental procedures used to examine the effects of MSCs in suppressing pyroptosis, upon exposure of macrophages to ITO-NPs, SiO_2_-NPs or MSU. (**b**) Cytometer analysis of MSCs shows cells negative for hematopoietic markers CD14, CD34 and CD45, but positive for MSC markers CD73, CD90, and CD105; 1 representative histogram in 4 for each marker is shown. (**c**) TEM of MSCs shows large cells with a clear cytoplasm and eccentric nucleus; 1 representative image in 3 is shown; original magnification ×1500. (**d**) MSCs appear on TEM as poorly capable of ITO-NP uptake (black arrow) at 12 h post-exposure; 1 representative image in 3 is shown; original magnification ×500. (**e**) TEM of MSCs co-cultured with MH-S or PMA-activated THP-1 cells exposed to ITO-NPs. TEM reveals tight cell-to-cell contacts (orange arrow) between MSCs and macrophages containing ITO-NP endosomes (black arrow); original magnification ×3000 and ×8000, 1 representative TEM image in 3 is shown. (**f**) MSCs co-cultured with MH-S cells exposed to ITO-NPs exhibited LDH release that was inversely proportional to the number of MSCs present. (**g**) MSCs effect on TNF-α release by MH-S cells exposed to ITO-NPs. (**h**) MSCs co-cultured with PMA-activated THP-1 cells exposed to ITO-NPs were tested for LDH release, and the release of LDH was inversely proportional to the number of MSCs present. (**i**) TNF-α release by PMA-activated THP-1 cells exposed to ITO-NPs in co-culture with MSCs. (**j**) MSCs co-cultured with PMA-activated THP-1 cells exposed to ITO-NPs were examined for IL-10 release. MSCs alone are represented as an empty green circle on the graph. Concentration of ITO-NPs was 500 μg/mL, and exposure time 16 h, unless stated otherwise. (**b–j**) n = 3–6 ± SEM, *p < 0.05. TEM abbreviations, Cyto = cytoplasm, N = nucleus and E = endosome.

Next, we examined whether mesenchymal stem cells (MSCs) could prevent both the inflammatory response and cell death in macrophages undergoing pyroptosis (Fig. 8a). MSCs possess immunosuppressive and regenerative properties capable of controlling inflammatory disorders such as graft versus host disease, Crohn’s disease and chronic obstructive pulmonary disease^25^,^26^,^38^. Here, primary MSCs were derived from adult human bone marrow mononuclear cells^39^. MSCs were expanded *in vitro* and established as CD14^-^CD34^-^CD45^-^ non-hematopoietic cells and phenotypically characterized as CD73^+^CD90^+^CD105^+^ cells (Fig. 8b). On TEM, MSCs were large with a clear cytoplasm containing an eccentric nucleus and were morphologically discernable from macrophages (Fig. 8c,e). MSCs co-cultured with MH-S or PMA-activated THP-1 cells exposed to ITO-NPs were analyzed by TEM. MH-S and PMA-activated THP-1 cells but not MSCs were capable of ITO-NP endocytosis (Fig. 8d,e). Furthermore, TEM of co-cultured MSCs and macrophages showed tight cell-to-cell interaction between MSCs and macrophages exhibiting endosomes containing ITO-NPs, without displaying plasma membrane rupture or apoptosis morphological features (Fig. 8e). Furthermore, MH-S cells exposed to ITO-NPs released a reduced amount of LDH, which was directly proportional to the number of MSCs present (Fig. 8f). As well, TNF-α secretion decreased significantly when MSCs were co-cultured with ITO-NP-exposed MH-S cells (Fig. 8g). Similar results were found for MH-S exposed to SiO_2_-NPs and MSU crystals on co-culture with MSCs (Supplemental Fig. S10a,b). Consistently, MSCs alleviated LDH release and TNF-α secretion resulting from exposure to ITO-NPs in PMA-activated THP-1 cells (Fig. 8h,i). Similar protective effect of MSCs was found in PMA-activated THP-1 cells upon exposure to SiO_2_-NPs or MSU crystals (Supplemental Fig. S10c,d). Concomitantly, we found a significant increase in secretion of the immunosuppressive cytokine IL-10 in PMA-activated THP-1 cells co-cultured with MSCs and exposed to ITO-NPs, SiO_2_-NPs or MSU compared to controls (Fig. 8j and Supplemental Fig. S10e). Therefore, MSCs possess potent ability to protect macrophages against both arms of pyroptosis – inflammation and cell death – induced by ITO-NPs, SiO_2_-NPs or MSU crystals and might favor tissue regeneration by increasing IL-10 secretion to further promote tissue healing^40^.

## Discussion

Cummings K. and colleagues revealed that occupational inhalation of InO and ITO particles cause respiratory diseases^1^. These respiratory diseases are characterized by a rapid evolution: the median age at diagnosis is 35 years old, with cases of premature death^1^,^41^,^42^. To provide further insights into the pathophysiology for respiratory diseases; we examined the inflammatory ability of ITO-NPs *in vivo* in mice and *in vitro* in AMs and monocyte-derived macrophages.

We first show that ITO-NP–caused peritonitis in mice depended on NLRP3 inflammasome. Resident macrophages first in contact with ITO-NPs may undergo cell death *via* cytolysis, which can have a considerable impact by inducing prolonged inflammation with tissue damage and fibrosis. Consistently, a mouse model of bleomycin-induced pulmonary fibrosis showed that ATP released from injured cells amplified NLRP3 inflammasome^43^. Previous studies have shown that extracellular ATP availability and subsequent activation of the P2×_7_R/Panx1 pathway predominantly enhanced neutrophil accumulation in inflammatory diseases^10^,^12^,^44^,^45^. This agrees with our results showing that blockade of NLRP3 inflammasome with glybenclamide in the ITO-NP–mediated peritonitis mouse model principally reduced the influx of neutrophils and IL-1β production.

Then, we examined the ITO-NP effects in AMs and monocyte-derived macrophages. Particularly, AMs exposed to ITO-NPs, SiO_2_-NPs, or MSU crystals provoked a regulated cell death (RCD) distinguished by cytolysis that depended on caspase1 activity; this RCD was identified as pyroptosis^16^. Necroptosis, which is a RIPK1–dependent and caspase1–independent RCD that resembles to pyroptosis, was not involved in mediating cell death of AMs exposed to ITO-NPs. Indeed, blockade of RIPK1 with NEC-1 failed to rescue AMs from cytolysis. As well, blockade of ROS with NAC in AMs exposed to ITO-NPs did not impede cytolysis, which demonstrates the major role of caspase1 in inducing pyroptosis. Furthermore, ITO-NPs and SiO_2_-NPs induced pyroptosis in AMs *via* scavenger receptor class-A (SR-A) CD204, and endocytosis of these particles was required for execution of pyroptosis. The blockade of CD204, but not of SR-A MARCO or SR-B CD36, reduced the cytolysis induced by ITO-NPs or SiO_2_-NPs in AMs. Thus, SR-A CD204 could have a role in AMs as an extracellular pattern-recognition receptor^46^,^47^,^48^, acting upstream of NLRP3 inflammasome, to detect and facilitate endocytosis of ITO-NPs like SiO_2_-NPs.

Further, endocytosis of ITO-NPs by macrophages results in NLRP3 inflammasome activation, resulting in the production of IL-1β, IL-1α and TNF-α, and cytolysis, both events – inflammation and cell death – being caspase1-dependent. Moreover, ITO-NP–induced pyroptosis was abrogated in PMA-activated THP-1 cells with knockdown of NLRP3, ASC or caspase1. Thus, NLRP3 inflammasome was indispensable in mediating caspase1-dependent pyroptosis in macrophages exposed to ITO-NPs. Concurrently, we have excluded other inflammasome pathways such as NLRP1, NLRC4, NLRP6 or AIM2^14^,^37^,^49^ participating in caspase1-dependent pyroptosis induced by ITO-NPs. In addition, PMA-activated THP-1 cells with caspase1 knockdown and ITO-NP exposure were unable to undergo pyroptosis. This finding excludes the implication of caspase4 or caspase5, caspases known for inducing pyroptosis independently to NLRP3 inflammasome and caspase1^50^,^51^, in the cell death of PMA-activated THP-1 cells with ITO-NP exposure. Altogether, it appears that pyroptosis induced in macrophages upon exposure to ITO-NPs is strictly dependent on endocytosis of the particles, NLRP3 inflammasome activation and caspase1 function. However, both NLRP3 and ASC knockdown THP-1 cells, with PMA activation and with ITO-NP exposure underwent a cell death with features of apoptosis. While PMA-activated THP-1 cells with NLRP3 or ASC deficiency and ITO-NP exposure did not secrete IL-1β/TNF-α or undergo cytolysis, cell viability has declined with an increase in AnnexinV^+^PI^−^ apoptotic cells. Moreover, TEM of PMA-activated THP-1 cells knockdown with NLRP3 or ASC and ITO-NP exposure exhibited morphological features of apoptosis, with endocytic cells showing membrane blebs and apoptotic bodies. This finding agrees with the hypothesis that NLRP3 inflammasome might play a role as a molecular switch in guiding inflammatory (i.e., pyroptosis) and non-inflammatory (i.e., apoptosis) RCD^52^,^53^.

Thus, controlling prolonged inflammation induced by xenogenous compounds implies targeting both inflammation and cell death^54^. Although inflammation can be impeded by directly targeting inflammatory pathways, cell death might still occur among immune cells, thus disorganizing the innate immunity^16^,^54^. Indeed, DEX moderates lung fibrosis in rodents^55^,^56^, and glucocorticoids are used as treatment for certain respiratory diseases^57^; however, the actual benefit of DEX is still poorly defined^58^. In our study, we examined DEX properties in the context of pyroptosis induced by ITO-NPs, SiO_2_-NPs and MSU crystals in macrophages. We ascertained that DEX inhibits the production of IL-1β and TNF-α in macrophages exposed to ITO-NPs, SiO_2_-NPs or MSU crystals. Yet, DEX did not neutralize caspase1-dependent cell death induced by ITO-NPs in macrophages. Consistently, in a model of *Mycobacterium tuberculosis* (MTb) infection of microglial cells, which vigorously activate caspase1 and result in IL-1β and TNF-α upregulation, pre-treating microglial cells with DEX and infection with MTb downregulated IL-1β and TNF-α transcription^59^. In contrast, DEX can increase NLRP3 transcriptional expression in macrophages^60^, rendering macrophages even more responsive to caspase1-dependent cell death. Agreeing with this, our findings indicate that blockade of inflammatory pathways by DEX or by circumventing NLRP3 inflammasome somehow did not protect macrophages exposed to ITO-NPs against cell death. Thus, we sought an approach with MSCs to control both arms of pyroptosis –inflammation and cytolysis while preventing apoptosis.

Here, we show that MSCs can prevent pyroptosis in alveolar macrophages and monocyte-derived macrophages induced by ITO-NPs, SiO_2_-NPs and MSU crystals. MSCs cooperate with macrophages containing endosomes with ITO-NPs likely *via* cell-to-cell contact as assessed by TEM, ultimately resulting in decreased pro-inflammatory cytokines release, cell death and enhanced IL-10 immunosuppressive cytokine production. Hence, IL-10 is critical for both reducing inflammation and favoring tissue healing^61^,^62^. Most importantly, MSCs reduced cytolysis of macrophages exposed to ITO-NPs without increasing apoptosis. The anti-apoptotic properties of MSCs towards immune and non-immune cells are well established^63^,^64^,^65^. Here, we show that MSCs possess potent anti-pyroptotic effect in macrophages.

Altogether, our findings indicate that respiratory diseases induced by ITO-NPs can be mediated NLRP3-ASC assembly and caspase1-dependent pyroptosis in macrophages, and identifies MSCs as promising therapeutic to suppress pyroptosis (Supplemental Fig. S11). These conclusions may be extended to other inflammatory disorders involving pyroptosis such as in silicosis and gout. Further investigations on the molecular pathways involved in MSCs for preventing pyroptosis in cells are critical to improve MSC-based therapeutic settings in inflammatory diseases and in regenerative medicine.

## Methods

### ITO-NPs, SiO_2_-NPs and MSU

Indium (III) oxide: Tin (IV) oxide (ITO) nanoparticles (ITO-NPs) are particles with size <50 nm (Sigma-Aldrich). Silicon (IV) oxide SiO_2_-NPs are particles with diameter <100 nm (Invivogen). Monosodium urate (MSU) is a solid crystal (Adipogen). ITO-NPs and SiO_2_-NPs apparent size were assessed on transmission electron microscopy. ITO-NPs and SiO_2_-NPs were used as particles in suspension, while MSU was soluble in complete medium. Complete medium consisted of RPMI 1640 (Sigma-Aldrich), 10% heat-inactivated FBS (Gibco), L-glutamin, streptomycin and penicillin (Sigma-Aldrich). Solution containing ITO-NPs were UV light-treated and absence of endotoxin-content were assessed on mouse splenocytes compared to stimulation with positive controls containing lipopolysaccharide endotoxins.

### Mouse peritonitis model

All animal experiments, including procedures, sampling and animal cares, were approved by the ethical committee of Kochi Medical School (Japan), in accordance with guidelines and regulations. Mice used in this study were purchased from SLC, Inc (Japan), and were allowed one week for acclimation to the animal facility (Kochi Medical School) before studies were initiated. Peritonitis was induced by injection of 2 mg of ITO-NPs in 1 mL PBS into the peritoneum cavity of male Balb/c mice (7–12 weeks). 6 h post-inoculation, mice were sacrificed and peritoneal cavities were washed with 1 mL of PBS. Peritoneal lavage fluids were centrifuged and supernatants were kept at −20 °C for ELISA. Repeated peritoneal lavage (5 times) with 1 mL PBS was performed to obtain peritoneal cells. Total peritoneal cells were used for flow cytometer analysis. Blockade of NLRP3 inflammasome was achieved by injecting i.p. 200 μL/mouse of a glybenclamide (Sigma-Aldrich) solution in PBS (Gly) representing a dose of 50 mg/kg, at least 45 min before ITO-NP injection.

### Cells

Peritoneal cells were from naïve Balb/c mice and seeded at 2 × 10^6^ cells/mL complete medium, allowed to adhere in culture dishes for 1 h in an incubator. Adherent cells were peritoneal macrophages assessed as CD11b^+^F4/80^+^cells by flow cytometer. Splenocytes were from spleen and bone marrow cells were obtained by flushing tibiae and femora of naïve Balb/c mice. Bone marrow–derived macrophages (BMDMs) were obtained by culture of bone marrow cells in a BMDM medium consisting of RPMI 1640, 10% FBS, 1% penicillin/streptomycin, 0.01% 2-β mercaptoethanol (Sigma-Aldrich), and 20 ng/mL of recombinant murine GM-CSF (Biolegend). After 4 days, BMDMs medium was replaced, and adherent cells were harvested after 8 days as BMDM. MH-S and THP-1 wild-type (WT), both from ATCC, were used as alveolar (mouse) and monocyte-like (human) macrophages. THP-1 cells knockdown in NLRP3, ASC and caspase1 were from Invivogen. Cells were cultured in RPMI 1640 complete medium. Hygromycin B selective antibiotic (Sigma-Aldrich) was used at 100 μg/mL for knockdown THP-1 cells. Lipopolysacharride (LPS) from *Escherichia coli* 0111:B4 (Sigma-Aldrich) was used at 100 ng/mL in some experiments. Phorbol 12-myristate 13-acetate (PMA) from Sigma-Aldrich, was used at 20 ng/mL to activate THP-1 cells; about 1 h before experiments.

### Mesenchymal stem cells (MSCs) and co-culture with macrophages

Approval for collecting procedures and use of human biological samples were obtained from the medical ethics committee at the University Hospital of Toulouse (France), with written informed consents obtained from all donors. Human marrow cells were from femoral heads adults undergoing orthopedic surgery. Mononuclear cells contained in marrow cells were isolated by Ficoll-Histopaque 1077 (Sigma-Aldrich), plated in culture flasks at 10^5^ cells/cm^2^, in α-MEM (Gibco), 10% heat-inactivated FBS, and 1 ng/mL human recombinant basic fibroblast growth factor (b-FGF, RD Systems). The culture was depleted of hematopoietic cells by successive medium removal made every other day. A confluent spindle/fibroblastic cell monolayer was obtained about 15 day after initial plating and were considered as MSCs passage 0 (P0). MSCs were used at P4 to P8 for phenotyping, characterization and co-culture experiments. MSC were allowed to adhere before co-culture with macrophages in RPMI 1640 complete medium. For TEM, M:MSCs co-culture were carried out with M:MSC of 2:1, M = 10^6^ cells in 6-well plate. For ELISA, M:MSCs co-culture experiments were carried out in 24-well plates at M:MSC of 2:1, M = 10^6^ cells. For LDH release, MSCs were co-cultured with macrophages at M:MSC 5:1, 2:1 and 1:1, M = 10^5^ cells in round-bottom 96-well plates.

### Pharmacological inhibitors and blockade with antibodies

Inhibitors (all from Sigma-Aldrich) and antibodies were added to cells at least 30 min before stimulation. Dexamethasone (DEX) was used at 100 ng/mL. Ac-Tyr-Val-Ala-Asp-chloromethylketone (Ac-YVAD), inhibitor of caspase1, was used at 10 μg/mL. Cytochalasin D (Cyto D) was used at 20 μM. Necrostatin-1 (NEC-1), an inhibitor of RIPK1 was used at 50 μM. N-Acetyl-(L)-cysteine (NAC) was used at 20 μg/mL. Purified monoclonal antibodies (mAbs) for blockade of MARCO (Clone ED31) and CD204 (Clone 2F8) were from AbD Serotec, and purified mAbs for CD36 (Clone JC63.1) were from Cayman Chemical. All mAbs were used at 2 μg/mL.

### Cell viability

Cells were plated at 10^5^ cells per well in a final volume of 200 μL. CCK-8 solution (Sigma-Aldrich) was added then plates were incubated for 4 h and read on a microplate reader (Multiskan, Thermo Scientific) at 450 nm. Data were obtained as OD and given as % of control, with control = cell in complete medium and, % of control = (OD sample/OD mean of control) ×100.

### [^3^H]-Thymidine incorporation

Cells were inoculated into round-bottom 96-well plates at 2.5 × 10^4^ cells per well in of 200 μL, stimulated under various conditions and allowed to incubate at 37 °C for 6 h, then pulsed with 0.1 μCi [^3^H]-Thymidine (Perkin-Elmer) for 18 h at 37 °C. Cells were harvested onto FilterMat A filters (Wallac) by using an automated cell harvester (Harvester 96, Tomtec). The FilterMat A was then dried and a solid scintillator (Meltlex, Perkin-Elmer) was used according to the manufacturer’s instructions. β-scintillation counts, counts per minute (c.p.m), of [^3^H]-Thymidine incorporated into DNA were determined with a MicroBeta reader (Wallac, Perkin-Elmer).

### [^51^Cr] Chromium release

Cells were labeled with [^51^Cr] (Perkin-Elmer) by incubating 10^6^ cells in RPMI 1640, 1% heat-inactivated FBS and 50 μCi of [^51^Cr] for 1 h at 37 °C. [^51^Cr]-labeled cells were washed 3 times with PBS and re-suspended in complete medium. Cells were inoculated in 96-well round-bottom plates, 2.5 × 10^4^ cells/well were stimulated in a volume of 200 μL and incubated at 37 °C for 6 h. Then, 30 μL of supernatants from each well were transferred into a scintillator-coated plate (Lumaplate, Perkin-Elmer) and incubated overnight at room temperature. [^51^Cr] was counted by β-scintillation, counts per minute (c.p.m) with use of a Microbeta reader (Wallac, Perkin-Elmer). Cells treated with 2% Tween 20 (Sigma-Aldrich) in distilled water represented a measure for maximal [^51^Cr] release, and cells in complete medium were a measure of spontaneous [^51^Cr] release. Results were given as raw c.p.m or as [^51^Cr]-release % of maximum release = (sample [^51^Cr]-release)-(spontaneous [^51^Cr]-release)/(maximal [^51^Cr]-release)-(spontaneous [^51^Cr]-release) ×100.

### LDH release

10^5^ cells/well were stimulated in round bottom 96 well-plates in a volume of 200 μL. 100-μL of supernatant from each well was transferred into flat-bottom 96-well plates, then catalyst and dye solutions were added (LDH Detection Kit, Takara). Then 100 μL HCl 1N/well were added, and plates were read on a microplate reader (Multiskan, Thermo Scientific) at 450 nm. Results are given as % of maximum release, with medium = no cells and TX-100 = cells + 1% Triton X-100, finally % of maximum release = (OD sample − OD medium)/(OD TX-100 − OD medium) ×100.

### Flow cytometry

Cells were stained in PBS with 1% FBS and Trustain FcX (Biolegend) and antibodies conjugated with FITC, PE or APC. Stained cells were fixed in PBS 1% FBS and 0.5% formaldehyde (Sigma-Aldrich). Mouse antibodies for CD11b (Clone M1/70), CD45 (Clone 30-F11), F4/80 (Clone BM8), Gr-1 (Clone RB6-8C5), or Ly-6G (Clone 1A8) were from Biolegend. Mouse antibodies for MARCO (Clone ED31) and CD204 (Clone 2F8) were from AbD Serotec. Mouse antibody for CD36 (Clone HM36) was from Biolegend. Human antibodies for CD90 (Clone DG3), CD105 (Clone 43A4E1), CD73 (Clone AD2), CD34 (Clone 581), CD45 (Clone 5B1) and CD14 (Clone M5E2) were from Becton Dickinson. Detection of phosphatidylserine (PS) was achieved with FITC-conjugated Annexin V, cells were concomitantly stained with a propidium iodide (PI), and reagents were from Sigma-Aldrich. Appropriate staining controls were performed, data were acquired on a flow cytometer (FACS Calibur, or LSR Fortessa BD Biosciences) and analyzed by FlowJo (Tree star Inc.) or Flowing (Turku Center).

### Transmission electronic microscopy (TEM)

Cellular samples were fixed in 0.9% NaCl, 1% glutaraldehyde (Wako) for 1 h at 4 °C. Cells were pelleted and re-suspended in 1 mL of a 37 °C pre-warmed 1.1% agarose solution (Bio-Rad). After centrifugation (3000 rpm at 37 °C), pellets were placed at 4 °C and cut into small specimens, washed in 5% sucrose PBS (Wako) and post-fixed with 5% sucrose, 1.5% osmic acid (Nisshin EM) in 100 mM phosphate buffer (Wako) pH 7.3, for 1 h at 4 °C. Post-fixed specimens were washed in 5% sucrose PBS and dehydrated in a graded series of ethanol (Wako) and propylene oxide (Wako), then embedded in epoxy resin EPON 812 (TAAB Laboratories). Ultrathin sections were obtained with an ultra-microtome (Leica EM UC7) and subjected to double staining with a uranyl-acetate (Wako) and lead-citrate (Nacalai Tesque). Samples were observed under a JEM-1400Plus Electronic Microscope (JEOL). TEM were analyzed by collecting images of at least 3 representative areas (RA) at magnification of ×500. One RA contained 10–20 nucleated cells. Then representative images were taken at higher magnifications.

### Total protein extraction and titration

Dry cell pellets were obtained after PBS washing and centrifugation of tested cells (1500 rpm, 8 min at 4 °C). Tris-HCl lysis buffer consisting of 50 mM Tris-HCl, 300 mM NaCl and 0.5% Triton X-100, pH 7.6, was added, 250 μL of lysis buffer/pellet. Cells were vortexed and kept for 1.5 h on ice. Cell lysates were sonicated with 3 pulses of 5 s, minimum power (Ultrasonic, UTH-80), and centrifuged at 16,000 g for 10 min at 4 °C. The cell extracts were placed into new test tubes. 10 μl of cell extracts was diluted into 990 μl PBS and used for determination of total protein by Bradford assay (Micro BCA, Thermo Scientific). Cell extracts were diluted with PBS from 1/4 to 1/8 and tested in ELISA for a given cytokine. ELISA data are given as picogram (intracellular cytokine) per milligram (pg/mg) total protein.

### ELISA

Samples for ELISA were supernatants or cell lysates of cells stimulated under conditions with cells plated at 10^6^ cells in 1 mL final volume per well, in 24-well plates. Cell culture supernatants or cell lysates or biological fluids from mice were assayed for mouse IL-1β, TNF-α and IL-10 (Mouse ELISA MAX Deluxe, Biolegend). Cell culture supernatants or cell lysates were assayed for human IL-1α , IL-1β, TNF-α and IL-10 (Human ELISA MAX Deluxe, Biolegend).

### Statistical analysis

Data are given as mean ± SEM, unpaired *t* test or one-way ANOVA with a multiple comparison test were performed using GraphPad Prism 5.0 (GraphPad Software, Inc). P < 0.05 was considered statistically significant.

### Ethics statement

Approval for collecting procedures and use for research purposes of bone-marrow samples were obtained from the medical ethics committee (CPP# DC-2012-1612) at the University Hospital of Toulouse (France), and written informed consents were obtained from all donors. The institutional animal care and use committee at Kochi Medical School (Japan) reviewed and approved all animal experiments in this study. Animal care, use, and treatment were strictly in compliance with guidelines and regulations.

## Additional Information

**How to cite this article**: Naji, A. *et al*. Endocytosis of indium-tin-oxide nanoparticles by macrophages provokes pyroptosis requiring NLRP3-ASC-Caspase1 axis that can be prevented by mesenchymal stem cells. *Sci. Rep.* **6**, 26162; doi: 10.1038/srep26162 (2016).

## Supplementary Material

Supplementary Information
