# Supplementary material for: Endocytosis of indium-tin-oxide nanoparticles by macrophages provokes pyroptosis requiring NLRP3-ASC-Caspase1 axis that can be prevented by mesenchymal stem cells
Source: Sci Rep. 2016 May 19;6:26162. doi: 10.1038/srep26162 (PMC4872131; doi:10.1038/srep26162)
Supplement: Supplementary Information [file srep26162-s1.pdf]

SUPPLEMENTARY INFORMATION

Endocytosis of indium-tin-oxide nanoparticles by macrophages provokes pyroptosis requiring NLRP3-ASC-Caspase1 axis that can be prevented by mesenchymal stem cells

Abderrahim Naji* [1,2], Basilua André Muzembo [2,5], Ken-ichi Yagyu [1,3], Nobuyasu Baba [1], Frédéric Deschaseaux [4], Luc Sensebé [4], Narufumi Suganuma [1,2]

[1]Center for Innovative and Translational Medicine (CITM). Kochi Medical School, Kochi University, Kochi, Japan.

[2]Department of Environmental Medicine, Kochi Medical School, Kochi University, Kochi, Japan.

[3]Science Research Center, Division of Biological Research, Life Sciences and Functional Materials, Kochi Medical School, Kochi University, Kochi, Japan

[4]STROMALab, Université de Toulouse, UMR 5273 CNRS, INSERM U1031, EFS Pyrénées-Méditerranée, Toulouse, France.

[5]Department of Epidemiology, Infectious Disease Control and Prevention, Institute of Biomedical and Health Sciences, Hiroshima University, Hiroshima, Japan.

*Correspondence should be addressed to A.N., email: najiab@kochi-u.ac.jp
